# Sylvatic Transmission of Chikungunya Virus among Nonhuman Primates in Myanmar

**DOI:** 10.3201/eid2812.220893

**Published:** 2022-12

**Authors:** Tierra Smiley Evans, Ohnmar Aung, Olivia Cords, Lark L. Coffey, Talia Wong, Christopher M. Weiss, Min Thein Maw, JoAnn Yee, Kodumudi Venkateswaran, Neeraja Venkateswaran, Peter Nham, Koen K.A. Van Rompay, Mary Kate Morris, Leo Oceguera, William Werthimer, Carl Hanson, Marc Valitutto, Kyaw Yan Naing Tun, Ye Tun Win, Wai Zin Thein, Susan Murray, Hlaing Myat Thu, Christine K. Johnson

**Affiliations:** University of California, Davis, California, USA (T. Smiley Evans, O. Aung, O. Cords, L.L. Coffey, T. Wong, C.M. Weiss, J. Yee, P. Nham, K.K.A. Van Rompay, C.K. Johnson);; Livestock Breeding and Veterinary Department, Yangon, Myanmar (M.T. Maw, Y.T. Win, W.Z. Thein);; Tetracore, Inc., Rockville, Maryland, USA (K. Venkateswaran, N. Venkateswaran);; California Department of Public Health, Richmond, California, USA (M.K. Morris, L. Oceguera, W. Werthimer, C. Hanson);; Smithsonian Institution, Washington, DC, USA (M. Valitutto, S. Murray); Department of Medical Research, Yangon (H.M. Thu)

**Keywords:** chikungunya virus, vector-borne infections, viruses, zoonoses, communicable diseases, Zika virus, Japanese encephalitis virus, Myanmar

## Abstract

Nonhuman primates living in proximity to humans increase risks for sylvatic arbovirus transmission. We collected serum samples from nonhuman primates in Hlawga National Park near Yangon, Myanmar, and detected antibodies against chikungunya (33%) and Japanese encephalitis (4%) viruses. Buffer zones between primate and human communities might reduce cross-species arbovirus transmission.

Several endemic and emerging arboviruses, such as chikungunya (CHIKV), Zika (ZIKV), and dengue (DENV) viruses, have evolutionary origins in nonhuman primates (NHPs) (*1*,*2*). These pathogens have adapted sylvatic to urban transmission cycles by using humans as amplifying hosts where NHPs are no longer required for virus maintenance. However, sylvatic arbovirus transmission cycles involving NHPs could act as sources of human infections, which would affect public health. NHPs could enable reemergence of arbovirus infections after immunity has waned following human–mosquito–human transmission. Sylvatic cycles can also provide selective environments where new viral strains can emerge.

CHIKV circulates in distinct enzootic, sylvatic transmission cycles in old world monkeys in the forests of sub-Saharan Africa (*2*). Limited data are available on sylvatic CHIKV transmission in Asia, but seroconversion has been detected in cynomolgus macaques (*Macaca fascicularis*), pig-tailed macaques (*M*. *nemistrina*), black-crested Sumatran langurs (*Presbytis melalophos*), and dusky leaf monkeys (*Presbytis obscura*) in Thailand (*3*,*4*), and virus has been isolated from long-tailed macaques in Malaysia (*5*). A sylvatic ZIKV lineage in Africa, infecting Cercopthecidae primate species, is known to circulate widely (*6*). The only positive ZIKV serology findings in primates in Asia have been in orangutans (*Pongo pygmaeus*) in Borneo, Malaysia, but those exposures were likely from an urban strain (*7*). Sylvatic DENV cycles occur in the forests of Malaysia in *Macaca* and *Presbytis* spp. monkeys (*8*) and also in West Africa; sylvatic DENV-2 circulates regularly between *Erythrocebus patas* monkeys and various *Aedes* spp. mosquitoes in Senegal (*8*). Seroprevalence of Japanese encephalitis virus (JEV) has been reported in cynomolgus monkeys, Japanese macaques (*M*. *fuscata*), green monkeys (*Chlorocebus sabaeus*), and pig-tailed macaques in several countries in Asia (*4*,*9*,*10*).

Myanmar is among the least studied but most heavily forested region in Asia, and CHIKV, ZIKV, DENV and JEV are highly endemic in humans. We investigated whether Myanmar peri-urban primates, living near the largest urban city of Yangon, are exposed to arboviruses of public health concern and could be sources of spillover or recipients of spillback of human pathogenic arboviral diseases.

## The Study

We collected specimens from 107 rhesus monkeys (*Macaca mulata*) and 12 pig-tailed macaques within Hlawga National Park, an open zoo and wildlife sanctuary in Myanmar’s Yangon region that covers 6.23 km^2^ (Figure). NHPs are free ranging within this park and have frequent opportunities for human contact. Serum samples were collected during October 2016–August 2017, which spanned 2 dry/wet seasons. We used a Luminex xMAP multiplex bead-based assay (Luminex Corp., https://www.luminexcorp.com) to simultaneously measure total IgG, IgA, and IgM against CHIKV E1 envelope protein, ZIKV nonstructural protein 1 (NS1), ZIKV envelope protein, DENV-1–4 NS1, JEV NS1, West Nile virus NS1, yellow fever virus NS1, and tickborne encephalitis virus NS1 (Appendix Table). We confirmed positive serum samples by using the plaque reduction neutralization test (Appendix). Conventional reverse transcription PCR targeting conserved regions of *Flavivirus* and *Alphavirus* spp. was performed to detect arbovirus viremia (Appendix).

**Figure:**
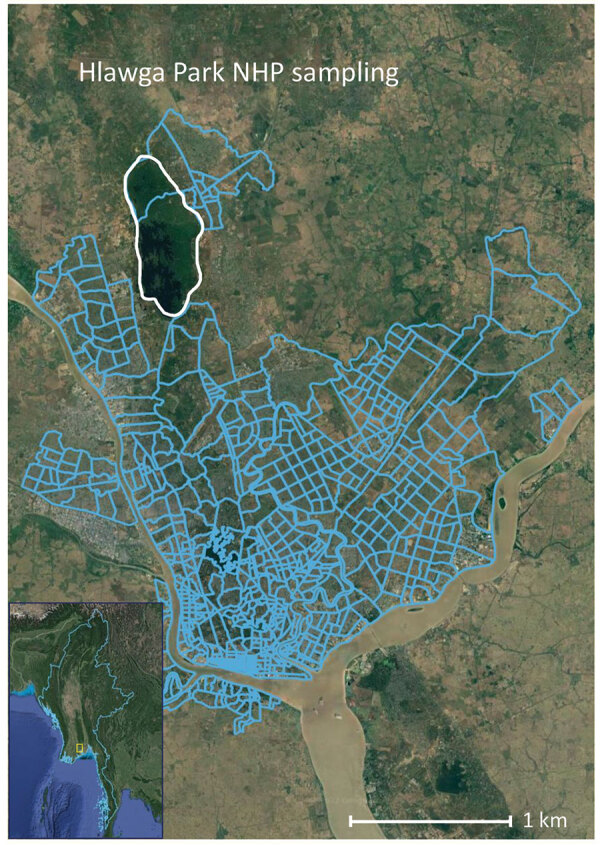
Hlawga National Park sampling site (white outline) in Yangon in study of sylvatic transmission of chikungunya virus among NHPs in Myanmar. Blue lines show the Yangon city wards south of the park. Inset shows location of Yangon in Myanmar (white box). NHP, nonhuman primate.

We identified virus-reactive antibodies among NHPs in Hlawga National Park, suggesting prior exposure to arboviruses, but we did not detect viruses by using PCR, suggesting absence of active infections. We found 33% (39/119) of NHPs were seropositive for CHIKV and 4% (5/119) were seropositive for JEV (Table); all serum samples were negative for ZIKV, West Nile virus, yellow fever virus, and tick-borne encephalitis virus. Using bivariate analysis, we showed specimens collected during the dry season were more likely to be seropositive for CHIKV (p = 0.05). Greater proportions of adult NHPs appeared to be seropositive for CHIKV; however, the difference was not statistically significant. We found no statistically significant associations between sex, age class, or species and specific arbovirus exposure. CHIKV and JEV in NHPs in Myanmar have not been reported, likely because of limited surveillance. Our findings extend the geographic range of potential sylvatic cycles for CHIKV to forests and peri-urban areas of Myanmar.

**Table:** Chikungunya and Japanese encephalitis virus prevalence during October 2016–August 2017 in Hlawga National Park in study of sylvatic transmission of chikungunya virus among nonhuman primates in Myanmar*

Characteristic	Chikungunya virus				Japanese encephalitis virus		
	No. positive†	No. negative†	Period prevalence		No. positive†	No. negative†	Period prevalence
Species							
*Macaca nemestrina* (pig-tailed macaque)	8	4	67 (35–90)		1	11	8 (0–38)
*Macaca mulatta* (rhesus macaque)	31	76	29 (21–39)		4	103	4 (1–9)
Sex							
M	20	43	32 (21–45)		3	60	5 (0–13)
F	19	37	34 (22–48)		2	54	4 (0–12)
Age class							
Adult	23	35	40 (27–53)		1	57	2 (0–9)
Subadult	16	45	26 (16–39)		4	57	7 (2–16)
Season							
Wet	25	69	27 (18–37)		4	90	4 (1–11)
Dry	14	11	56 (35–76)		1	24	4 (0–20)
Overall	39	80	33 (24–42)		5	114	4 (1–10)

*Values are no. or % (95% binomial exact CI). †No. positive or negative serum samples according to the total Ig detected by multiplex bead-based assay. Cutoff values were determined according to the lowest median fluorescence intensity value detected by a positive plaque reduction neutralization test.

Our results indicate that NHPs were exposed to CHIKV during a period with no or limited human–mosquito–human transmission, suggesting that seropositive samples resulted from sylvatic exposures. IgG against CHIKV E2 protein can be detected up to 21 months postinfection (*11*). If similar kinetics occur in NHPs and extend to the E1 protein, NHP exposures to CHIKV could have occurred during 2013–2014 or earlier. However, in 2017, we detected CHIKV antibodies in NHPs that were <5 years of age, indicating exposure during an interepidemic period. Human cases of CHIKV were not reported by the Myanmar Ministry of Health during 2011–2018 (*12*), and CHIKV outbreaks are not commonly underreported because a large proportion of infected persons have indicative arthritic manifestations. In 2019, health officials reported widespread outbreaks of CHIKV in Mandalay, Nay Pyi Taw, Kachin State, Tanintharyi, and Yangon regions of Myanmar, indicating reemergence of the virus (*12*).

We studied an NHP population that lived in a forested area outside of Yangon and could have played a role in the reemergence of CHIKV in humans. The large proportion of NHPs that were exposed indicated the virus was circulating among sylvatic mosquitos and primates in this park. The absence of reported human infections during the potential period of NHP infection suggested that spillover from humans to NHPs via mosquitoes was unlikely. *Aedes aegypti* and *A*. *albopictus* mosquitoes, the two primary urban vectors of CHIKV, are also known to feed almost exclusively on humans in the region, providing further evidence that NHP exposures to CHIKV in our study population were of sylvatic origin (*13*).

Our findings indicate that JEV is circulating at the periphery of Yangon, and NHPs can be occasional incidental hosts. JEV is endemic in Myanmar, particularly in the Yangon region (*14*). NHPs are not thought to be potential reservoirs, but are dead-end hosts; they produce a low viremia that cannot subsequently infect mosquitoes (*1*). Low levels of viremia produced in experimental studies and sylvatic cycles involving waterfowl or pigs are well documented. Furthermore, JEV is transmitted to humans by infected *Culex* spp. mosquitoes (most commonly *Culex tritaeniorhynchus*), which feed on many mammals in the region (*15*), making it more plausible that NHPs could be incidental targets of this mosquito species.

We did not confirm NHP exposure to DENV or ZIKV. We identified positive samples by using the Luminex assay, but those samples tested negative when the plaque reduction neutralization assay was used for confirmation. DENV is endemic in humans in Myanmar, and our findings indicate that spillback of urban DENV strains to NHPs is not common in this region or was not detected in our sample size. Given the limited knowledge of the scope of human ZIKV circulation in Myanmar and lack of entomological data, further research is needed to examine potential sylvatic ZIKV cycles among NHPs in Asia.

## Conclusions

Our study demonstrates the importance of conducting surveillance of peri-urban primates in regions of high arbovirus transmission and the need for less invasive methods that improve feasibility. Future research on molecular epidemiology of arboviruses in humans, NHPs, and mosquitoes is needed to confirm whether exposures result from potential sylvatic cycles of ongoing transmission or spillback events from urban strains. A heightened awareness of new CHIKV outbreak potential in humans living near NHPs in Hlawga National Park is warranted. Buffer zones between parks and human settlements might reduce future cross-species arbovirus transmission.

Appendix Additional information for sylvatic transmission of chikungunya virus among nonhuman primates in Myanmar.
