# Supplementary material for: Sylvatic Transmission of Chikungunya Virus among Nonhuman Primates in Myanmar
Source: Emerg Infect Dis. 2022 Dec;28(12):2548–51. doi: 10.3201/eid2812.220893 (PMC9707571; doi:10.3201/eid2812.220893)
Supplement: Appendix — Additional information for sylvatic transmission of chikungunya virus among nonhuman primates in Myanmar. [file 22-0893-Techapp-s1.pdf]

Article DOI: https://doi.org/10.3201/eid2812.220893

Sylvatic Transmission of Chikungunya Virus among Nonhuman Primates in Myanmar

Appendix

Additional Methods

All animal sampling protocols were reviewed and approved by the Institutional Animal Care and Use Committee at the University of California Davis (approval no. 19300) and the Livestock Breeding and Veterinary Department, Ministry of Agriculture, Livestock and Irrigation, Yangon, Myanmar. We defined the dry season in Myanmar as November through May and the wet season as June through October. We defined a subadult nonhuman primate (NHP) as being fully independent and appearing sexually mature but not fully physically mature (e.g., <full adult size). We defined an adult NHP as having secondary sexual characteristics, adult size, and being sexually mature.

Arbovirus Multiplex Assay

We used the FlexImmArray Arbovirus (19-Plex) NHP IgGMA Antibody Test (Tetracore, Inc., https://www.tetracore.com) to test NHP serum samples. This multiplex bead-based assay uses Luminex xMAP technology and comprises 13 different arbovirus-specific target proteins that detect total IgG, IgA, and IgM antibodies against 10 different viruses (Appendix Table). The test also includes 6 internal controls to monitor assay performance at each step. The multiplex assay uses magnetic microspheres coupled with unique recombinant proteins specific for different viral targets to capture antibodies from NHP serum samples. We mixed 25 μL of each serum sample (diluted 1:250) with 25 μL of antigen-coated microspheres in wells of a 96-well plate. We placed the plates on a shaker set at 800 rpm and incubated for 30 min at room temperature in the dark. The antigen-specific antibodies were immobilized on the microspheres, and we washed unbound material 4× with 200 μL wash buffer. We detected the antigen-captured antibodies by incubating with 25 μL of phycoerythrin-labeled antibodies against NHP IgG, IgA, and IgM conjugates for 30 min at room temperature in the dark on a shaker set at 800 rpm. Unbound material in the plate was washed 4× with 200 μL assay wash buffer. After the final

wash, we resuspended the microspheres in 150 µL of wash buffer and analyzed them by using a Luminex MAGPIX instrument and xPONENT software (Luminex Corp., https://www.luminexcorp.com).

We initially tested serum samples at a 1:250 dilution. Subsequently, we tested suspected positive samples by using serial dilutions of 1:500 and 1:1000. Data were evaluated as the median fluorescence intensity (MFI) of each bead set minus the MFI of the non-specific binding control. To determine initial cutoff values, we evaluated MFIs minus the non-specific binding control for all samples and conservatively set the cutoff values for suspected positives at 3 SD above the mean of the 6 lowest sample values. We confirmed a subset of suspected positive samples identified in the multiplex bead-based assay by using plaque reduction neutralization tests (PRNTs). Cutoff values were subsequently adjusted upwards according to the lowest MFI value at which a positive PRNT was detected.

Plaque Reduction Neutralization Assays

We tested suspected positive serum samples (determined by Luminex xMAP arbovirus assays) for serum neutralizing antibodies by using PRNTs. We heat-inactivated the serum samples at 56°C for 30 min and used the following protocols for each virus.

CHIKV

We used standard plaque methods for CHIKV as previously described (*1*). In brief, we serially diluted serum samples 2-fold in phosphate-buffered saline (PBS) from 1:10 through 1:320 and incubated the samples for 1 h at 37°C with 30 µL of 1600 PFU/mL wild-type CHIKV-06.049 infectious clone that was isolated from a patient in Réunion Island, France (*2*). We grew African green monkey kidney cells (Vero cells) to confluence in 12-well cluster plates, then incubated the cells with 160 µL of serially diluted specimen-virus mixture. After 1 h, we overlayed the cells with 0.5% agarose in Dulbecco’s Modified Essential Medium containing 5% fetal bovine serum and incubated for 72 h. We fixed the cells in 4% formaldehyde and removed agarose plugs. We stained the plaques for 10 min with 0.05% w/v crystal violet in 20% ethanol, then washed with cold water. We measured PRNT titers by manually counting plaques according to the serum dilution, where >80% reduction in the number of plaques ($PRNT_{80}$) compared with negative controls was observed. We considered a $PRNT_{80}$ titer of ≥10 as positive for CHIKV neutralizing antibodies (*3*,*4*).

DENV1–4

We used standard plaque methods as described previously with the following modifications. We diluted suspected DENV 1–4-positive serum samples 2-fold in Dulbecco's Modified Essential Medium from 1:10 through 1:320 and incubated the samples for 1 h at 37°C with 60 µL (1:1 ratio) of 100 PFU/µL DENV 1, 2, 3, or 4. We inoculated Vero cells with 100 µL of the serum-virus mixture and incubated for 1 h at 37°C. We overlayed the cells with 1% agarose prepared in 2x solution containing lactalbumin hydrolysate, yeast extract, Earle's Balanced Salt Solution, fetal bovine serum, gentamicin sulfate, fungizone, and 3% sodium bicarbonate. We incubated the cells for 96 h, then added a second overlay of 1% agarose in the 2x solution containing 3% sodium bicarbonate and 3% neutral red and incubated for 24 h at 37°C. We used human convalescent serum samples (BEI Resources, https://www.beiresources.org) as positive controls for DENV 1–4. We used suspected negative NHP serum samples (determined by Luminex xMAP arbovirus assays) as negative controls.

JEV and ZIKV

Standard plaque methods were used as described previously for CHIKV with the following modifications. We diluted serum samples 2-fold from 1:10 through 1:320 in 0.75% bovine serum albumin prepared in PBS. We mixed serum sample dilutions at a 1:1 ratio with 100 PFU of Japanese encephalitis virus strain SA14–14–2 (for JEV) or Zika virus strain PRVABC 59 (for ZIKV) that were diluted in bovine serum albumin in PBS and incubated the mixtures for 1 h at 37°C. We inoculated confluent monolayers of Vero CCL81 cells with 100 µL of each serum-virus mixture in 6-well plates and incubated at 37°C in a 5% CO_2 incubator for 1 h. After incubation, we overlayed the cells with 3 mL 0.6% Oxoid agarose prepared in Minimum Essential Medium containing 4 mmol/L L-glutamine, 50 µg/mL penicillin-streptomycin, 0.25 µg/mL amphotericin B, and 0.01% diethylaminoethyl-dextran. We incubated the plates at 37°C in 5% CO_2 for 3 d, then added a second overlay with 3 mL 1% Oxoid agar containing 0.3% neutral red in Minimum Essential Medium and incubated the cells for an additional 48 h. We included JEV antibody in polyclonal mouse immune ascites fluid as a positive control and a previously tested human serum sample positive for neutralizing antibodies against ZIKV. We also included negative control serum, cell controls, and a virus back-titration to verify virus PFU input. We counted individual plaques with the aid of a lightbox and compared those data with

virus control wells (no neutralizing plasma added). JEV, ZIKV, and JEV mouse immune ascites fluid were kindly provided by the World Reference Center for Emerging Viruses and Arboviruses (https://www.utmb.edu).

PCR Assays for Flaviviruses and Alphaviruses

We processed samples to detect viruses by using consensus PCR, which enables the universal amplification of sequences from viruses within a given family or genus and subsequent discernment of viral strains. We extracted total nucleic acids from whole blood by using Direct-zol RNA Miniprep kits (Zymo Research, https://www.zymoresearch.com) according to the manufacturer's instructions. We reverse transcribed total RNA into cDNA by using SuperScript III (Thermo Fisher, https://www.thermofisher.com) following the manufacturer's instructions and used 2 assays to detect flaviviruses and alphaviruses as described previously (*5*,*6*). As a positive control, we used synthetic DNA constructs containing a T7 promoter sequence that could be transcribed into RNA for reverse transcription-based assays.

Statistical Analyses

To determine associations between NHP demographic and seasonal variables, we evaluated correlations between all variables to assess potential confounding factors. We used chi-square or Fisher exact tests as appropriate to determine associations between specific arbovirus exposure and demographic and seasonal variables. We considered $p<0.05$ to be statistically significant. We then used multivariable logistic regression to assess the association between demographic and seasonal factors that were significant by bivariate analysis. We included variables if they improved model fit according to the likelihood ratio test ($p<0.1$) and minimized the Akaike information criterion. We assessed overall model fit by using the Hosmer-Lemeshow goodness-of-fit test. We performed all statistical analyses by using R, version 3.6.1 (The R Project for Statistical Computing, https://www.r-project.org).

References

1. Weiss CM, Liu H, Riemersma KK, Ball EE, Coffey LL. Engineering a fidelity-variant live-attenuated vaccine for chikungunya virus. NPJ Vaccines. 2020;5:97. PubMed https://doi.org/10.1038/s41541-020-00241-z

2. Coffey LL, Vignuzzi M. Host alternation of chikungunya virus increases fitness while restricting population diversity and adaptability to novel selective pressures. J Virol. 2011;85:1025–35. PubMed https://doi.org/10.1128/JVI.01918-10

3. Yoon IK, Alera MT, Lago CB, Tac-An IA, Villa D, Fernandez S, et al. High rate of subclinical chikungunya virus infection and association of neutralizing antibody with protection in a prospective cohort in the Philippines. PLoS Negl Trop Dis. 2015;9:e0003764. PubMed https://doi.org/10.1371/journal.pntd.0003764

4. Nitatpattana N, Kanjanopas K, Yoksan S, Satimai W, Vongba N, Langdatsuwan S, et al. Long-term persistence of chikungunya virus neutralizing antibodies in human populations of north eastern Thailand. Virol J. 2014;11:183. PubMed https://doi.org/10.1186/1743-422X-11-183

5. Moureau G, Temmam S, Gonzalez JP, Charrel RN, Grard G, de Lamballerie X. A real-time RT-PCR method for the universal detection and identification of flaviviruses. Vector Borne Zoonotic Dis. 2007;7:467–77. PubMed https://doi.org/10.1089/vbz.2007.0206

6. Sánchez-Seco MP, Rosario D, Quiroz E, Guzmán G, Tenorio A. A generic nested RT-PCR followed by sequencing for detection and identification of members of the alphavirus genus. J Virol Methods. 2001;95:153–61. PubMed https://doi.org/10.1016/S0166-0934(01)00306-8

Appendix Table. Details of the 19-plex microsphere mixture used in the arbovirus multiplex assay in study of sylvatic transmission of chikungunya virus among nonhuman primates in Myanmar*

Analyte no.	Microsphere region	Target antigen
1	26	Zika NS1, antigen 1
2	28	Zika NS1, antigen 2
3	30	Zika Env
4	27	DENV1 NS1
5	35	DENV2 NS1
6	37	DENV3 NS1
7	48	DENV4 NS1
8	39	WNV NS1
9	42	JEV NS1
10	43	YFV NS1
11	44	TBEV NS1
12	45	CHIKV E1, antigen 1
13	46	CHIKV E1, antigen 2
14	47	Instrument control
15	54	Nonspecific binding control
16	51	Human IgM control
17	52	Human IgG control
18	55	Human IgG, IgM, IgA control
19	53	Fluorescent reporter control

*We used the FlexImmArray Arbovirus (19-Plex) NHP IgGMA Antibody Test (Tetracore, Inc., https://www.tetracore.com) to test serum samples from nonhuman primates. CHIKV, chikungunya virus; DENV, dengue virus; E1, envelope protein 1; Env, envelope protein; Ig, immunoglobulin; JEV, Japanese encephalitis virus; NS1, nonstructural protein 1; TBEV, tickborne encephalitis virus; WNV, West Nile virus; YFV, yellow fever virus.
